# Habituation of glomerular responses in the olfactory bulb following prolonged odor stimulation reflects reduced peripheral input

**DOI:** 10.3389/fnmol.2015.00053

**Published:** 2015-09-23

**Authors:** M. Cameron Ogg, Mounir Bendahamane, Max L. Fletcher

**Affiliations:** Department of Anatomy and Neurobiology, University of Tennessee Health Science CenterMemphis, TN, USA

**Keywords:** calcium imaging, GCaMP2, olfactory bulb, glomerular layer, habituation

## Abstract

Following prolonged odor stimulation, output from olfactory bulb (OB) mitral/tufted (M/T) cells is decreased in response to subsequent olfactory stimulation. Currently, it is unclear if this decrease is a function of adaptation of peripheral olfactory sensory neuron (OSN) responses or reflects depression of bulb circuits. We used wide-field calcium imaging in anesthetized transgenic GCaMP2 mice to compare excitatory glomerular layer odor responses before and after a 30-s odor stimulation. Significant habituation of subsequent glomerular odor responses to both the same and structurally similar odorants was detected with our protocol. To test whether depression of OSN terminals contributed to this habituation, olfactory nerve layer (ON) stimulation was used to drive glomerular layer responses in the absence of peripheral odor activation of the OSNs. Following odor habituation, in contrast to odor-evoked glomerular responses, ON stimulation-evoked glomerular responses were not habituated. The difference in response between odor and electrical stimulation following odor habituation provides evidence that odor response reductions measured in the glomerular layer of the OB are most likely the result of OSN adaptation processes taking place in the periphery.

## Introduction

Olfactory sensory neurons (OSNs) in the nasal epithelium expressing the same type of olfactory receptor project to glomeruli in the olfactory bulb (OB; Mori et al., 1999; Feinstein and Mombaerts, 2004), a dense cluster of dendrites from interneurons and output mitral/tufted (M/T) cells. Because odors bind differentially to the olfactory receptors, each odor generates a unique pattern of glomerular activation in the bulb (Mori et al., 1999, 2006). These patterns can be visualized at either the presynaptic OSN input level or at the postsynaptic M/T cell level *in vivo* using various imaging methods (Pain et al., 2011; Fletcher and Bendahmane, 2014) and in some cases can reflect real time changes in responsivity following changes in odor input.

One such change, habituation, is the process by which animals decrease their responses to repeated or continually present stimuli (Wilson and Linster, 2008; Rankin et al., 2009). In the olfactory system, short-term habituation is likely primarily driven by a reduction of neuronal responsivity at several stages along the olfactory pathway from the periphery to the cortex (Dalton, 2000; Zufall and Leinders-Zufall, 2000; Wilson and Linster, 2008; Reisert and Zhao, 2011). Numerous studies have probed adaptation of OSN responses (Zufall and Leinders-Zufall, 2000; Reisert and Zhao, 2011) and M/T cell OB output (Wilson, 2000; Best and Wilson, 2004; Chaudhury et al., 2010). However, olfactory information is processed throughout the layers of the OB, including via inhibitory networks within the glomerular layer (Wachowiak and Shipley, 2006; Nagayama et al., 2014). Yet, few studies have addressed the impact of habituating odor stimulation on odor responses in the glomerular layer of the OB (Schafer et al., 2005; Lecoq et al., 2009).

Similarly to OSN and M/T cell output responses, these studies found glomerular layer response decreases with prolonged odor exposure or brief, very strong odor stimulations (Schafer et al., 2005; Lecoq et al., 2009). However, these studies both relied on recording methods that reflect the total activity of the glomerular circuit [functional magnetic resonance imaging (fMRI; Schafer et al., 2005) and local field potential recordings (Lecoq et al., 2009)] that cannot differentiate excitatory output responses from inhibitory interneuronal responses. Further, the extent to which this reduction reflects decreased input from OSNs, as suggested by a recent study (Lecoq et al., 2009), or a reduction in responsiveness of OB neurons is still unclear.

To address these questions about olfactory habituation in the glomerular layer, we measured glomerular responses before and after prolonged odor exposure in anesthetized transgenic mice expressing the fluorescent calcium indicator GCaMP2 in M/T and excitatory juxtaglomerular (JG) cells (Díez-García et al., 2005; Fletcher et al., 2009). We assessed glomerular responses to the same odorant (self-habituation) and to structurally similar, representationally overlapping odorants (cross-habituation) and compared them to that of M/T cell output responses reported previously (Wilson, 2000). To dissect the role of OSN adaptation in post-synaptic glomerular habituation, we also compared post-habituated odor-driven responses to responses driven by olfactory nerve layer (ON) electrical stimulation (Fletcher et al., 2009).

We found that glomerular odor responses to both the habituating odor (self-habituation) and to an odor that is structurally similar to the habituating odor (cross-habituation) decreased following a 30 s continuous odor pulse. At the moderate odor concentrations used in this study, neural response changes following self-habituation were relatively uniform across glomeruli regardless of initial response intensity. Therefore, the glomerular representation (spatial map and relative intensity) of the habituated odor was unchanged. In contrast, post habituation ON stimulation-evoked glomerular responses displayed little habituation. The difference in glomerular habituation between odor and electrical stimulation provides evidence that the odor response reductions measured in the OB are most likely the result of OSN adaptation processes taking place in the periphery and not a consequence of adaptation of the OSN-M/T synapse.

## Materials and Methods

### Animals and Surgery

Experiments were performed using 20 adult transgenic male and female mice expressing the green fluorescent Ca^2+^ indicator GCaMP2 under the Kv3.1 potassium channel promoter (Díez-García et al., 2005). Under this promoter, GCaMP2 is expressed in M/T cells and a subpopulation of JG cells (Fletcher et al., 2009). Mice were anesthetized with urethane (2 mg/kg, i.p.) and given an injection of methyl scopolamine (0.05 mg/kg, i.p) to prevent nasal congestion. Mice were secured in a custom stereotaxic apparatus (Narishige) with a heating pad underneath to maintain body temperature. To create an imaging window, a skin incision was made over the dorsal surface of the mouse head and the bone overlying the OBs was thinned with a dental drill. In cases in which electrical stimulation was used, part of the bone was removed after thinning. In some cases lidocaine was applied to the bulb through a small incision in the dura. A dental-cement well was built around the olfactory bulbs and filled with Ringer’s solution. During imaging sessions, animals were freely breathing and the respiratory rate was monitored from the respiratory oscillation observed in the odor-evoked GCaMP2 odor-evoked signal. All animal care protocols were approved by the University of Tennessee Institutional Animal Care and Use Committee.

### Odorant Presentation

Odors [2-hexanone, 2-heptanone, and ethyl butyrate (Sigma-Aldrich)] were delivered using a flow-dilution olfactometer previously described (Fletcher et al., 2009). Separate flow controllers for the clean air and the pure odorant vapor were used to mix the flow streams at the end of the odor delivery system to achieve an approximate concentration of 0.25, 0.5, or 0.75% saturated vapor (s.v.) at a flow rate of 0.7 L/min. The odor concentration used for each animal was a concentration that activated discrete, stable glomeruli.

### Olfactory Nerve Stimulation

For olfactory nerve layer electrical stimulation (ONS), a single current pulse (2 ms, 45–100 μA) was delivered to the OB dorsal surface using a bipolar tungsten electrode (World Precision Instruments). This method has been shown previously to evoke increased glomerular GCaMP signals via synaptically driven activity and is not a result of direct electric current stimulating glomerular postsynaptic dendrites (Fletcher et al., 2009). Further, topical application of the Na^+^ channel blocker lidocaine onto the OB completely blocked all ONS driven glomerular activity (see “Results” Section).

### Experimental Protocol

#### Experiment 1: Habituation Timeline

For control trials, odor pulse duration was 1 s with an inter-stimulus interval of at least 2 min. For the habituation trial, odor pulse duration was 30 s. For post-habituation trials, odor pulse duration was 1 s and the inter-stimulus interval varied.

#### Experiment 2: Cross-Habituation

For control trials, odor pulse duration was 1 s with an inter-stimulus interval of at least 2 min. Two-hexanone (C6) was presented during the 30-s habituation trial. Two-heptanone (C7) was given 30 s post-habituation and C6 was given 1 min post-habituation. We waited at least 10 min for the animals to recover from the first habituation, established new baseline responses for the two odors, and repeated the experiment with C7 as the habituating odor.

#### Experiment 3: ON-Stimulation

For control trials, odor pulse duration was 1 s and ONS duration was 2 ms with an inter-stimulus interval of at least 2 min. For the habituation trial, odor pulse duration was 30 s. For post-habituation trials, odor pulse duration was 1 s, ONS duration was 2 ms, and the inter-stimulus interval varied. Post-habituation trials occurred within 1 min following the odor habituation trial.

### Optical Imaging and Analysis

Imaging was performed using a Scientifica Slicescope equipped with a 10 × (0.3 NA) Olympus objective. The dorsal OB was illuminated with a LED light source centered at 480 nm. GCaMP2 signals were band-pass filtered with a Chroma emission filter (HQ535/50) and collected using a CCD camera at 25 Hz (NeuroCCD-SM256, Redshirt Imaging). Maps of stimulus-evoked spatial activity were generated by first correcting for photo-bleaching and then spatially low-pass filtered as described previously (Fletcher et al., 2009). The stimulus-evoked change in fluorescence (ΔF) was calculated by subtracting the average of five frames immediately preceding stimulus onset from the average of five frames centered on the peak of the response generated by the first respiration or electrical stimulation. Glomerular response amplitude (ΔF/F) was calculated by dividing the stimulus-evoked change in fluorescence by the resting fluorescence. For quantitative analysis, discrete glomeruli were visually identified and the response amplitude was measured from a ROI (2 × 2 pixel average) at the center of each (Fletcher et al., 2009). The response of each glomerulus was averaged across control trials. A glomerulus was considered to respond if its mean ΔF/F response to a stimulus was greater than the background ΔF/F signal. Background signal was defined as the mean ± 2 SD ΔF/F value obtained from adjacent regions containing no glomerular activity (Fletcher, 2011). Habituation was measured by dividing the post-habituation response of each glomerulus by its average control response. To identify overlapping glomeruli in both the cross-habituation and ONS experiments (i.e., glomeruli that respond to both odors or to both odor and ONS), ROIs were placed at the center of all glomeruli activated by either odor delivery or ONS for each animal. Glomeruli that responded significantly, as defined above, to both stimuli were defined as shared and were pooled across animals for analysis (Fletcher, 2011).

### Statistical Analysis

Statistical analyses were performed using Prism 5.0 software (Graphpad). Values are expressed as mean normalized response ± SEM (unless otherwise indicated). Data were compared using one sample *t*-test, paired *t*-test, one-way ANOVA, and repeated measures ANOVA (Dunnett’s test and Tukey’s test *post hoc* analyses were performed when appropriate). Statistical significance was defined as *p* < 0.05.

## Results

**Figure 1:**
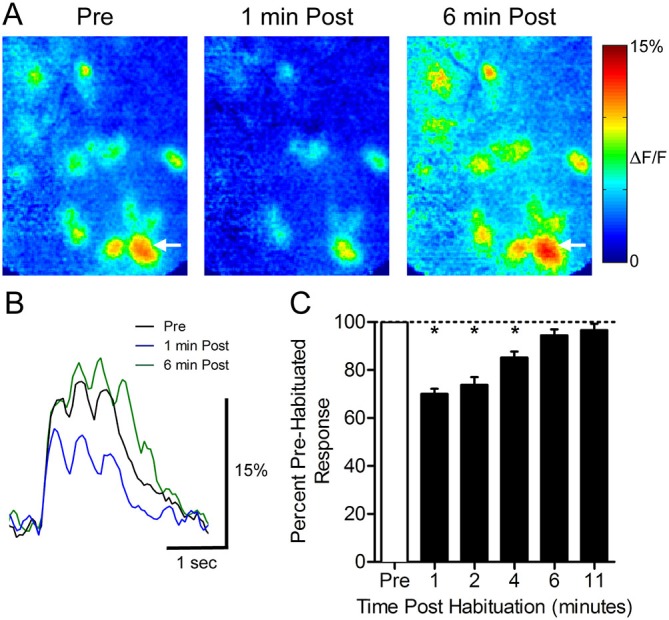
**Thirty seconds odor exposure decreases subsequent glomerular responses to that odor for several minutes. (A)** Pseudo-color glomerular responses to 2-heptanone (0.5% s.v.) at 10× magnification. One minute after the habituating odor exposure (1 min Post), glomerular responses are decreased from their baseline (Pre). After 6 min, the responses have recovered (6 min Post). **(B)** GCamp2 fluorescence traces from the glomerulus indicated by arrows in **(A)**. **(C)** The timeline of recovery from habituation. Mean normalized glomerular responses for all animals were reduced for several minutes post exposure. Error bars indicate SEM. *p < 0.05.

To determine how a 30-s odor exposure impacts subsequent glomerular responses to that odor, we measured glomerular responses to 1-s odor pulses before and after a single prolonged exposure in ten animals (Figure 1). Following the habituation trial, the mean normalized glomerular responses changed (ANOVA: *F*_(5,395)_ = 37.03, *p* < 0.0001), and *post hoc* tests showed significant reduction from baseline responses 1 min (70.1 ± 2.1%, *n* = 95), 2 min (73.8 ± 3.3%, *n* = 61), and 4 min (85.2 ± 2.5%, *n* = 49) post exposure (Figure 1C). Mean responses at 6 min (94.5 ± 2.5%, *n* = 61) and 11 min (96.6 ± 2.7%, *n* = 40) post-habituation were not significantly different from the baseline, indicating that recovery had occurred by 6 min.

**Figure 2:**
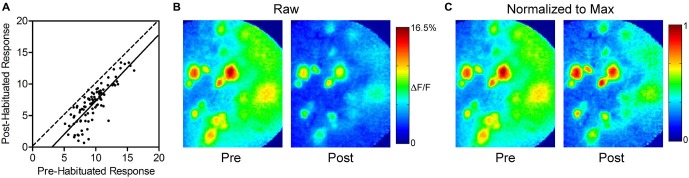
**Thirty seconds odor exposure uniformly decreases subsequent glomerular responses, regardless of initial intensity. (A)** Responses of each glomerulus before and 1 min after the habituating odor exposure are plotted against each other. The dashed line has a slope of unity. The solid best-fit line is parallel to the line with a slope of unity, indicating that glomeruli maintain their relative odor responses following habituation. The downward shift of the line reflects the effect of habituation across glomeruli. **(B)** Psuedo-color glomerular responses to 2-heptanone (0.5% s.v.) at 10× magnification before (Pre) and after (Post) the habituating odor exposure. **(C)** The glomerular responses shown in **(B)**, normalized to the maximum glomerulus in each representation, illustrating that glomeruli maintain their relative odor responses following habituation, as discussed in **(A)**.

To determine if there was an effect of response intensity on the amount of habituation, we compared the responses of each glomerulus before and 1 min after habituating odor exposure (Figures 2A,B). Linear regression analysis yielded a best-fit line with a slope (1.1 ± 0.1, not significant) showing that habituation has a uniform effect regardless of response intensity, and does not disproportionately decrease the response of either strongly or weakly responding glomeruli. The uniform reduction leaves relative glomerular response magnitudes of individual odor representations intact following prolonged odor stimulation. This effect is illustrated in Figure 2C, which highlights the similarity of pre- and post-habituation odor maps when they are normalized to the maximally responding glomerulus in each odor representation.

**Figure 3:**
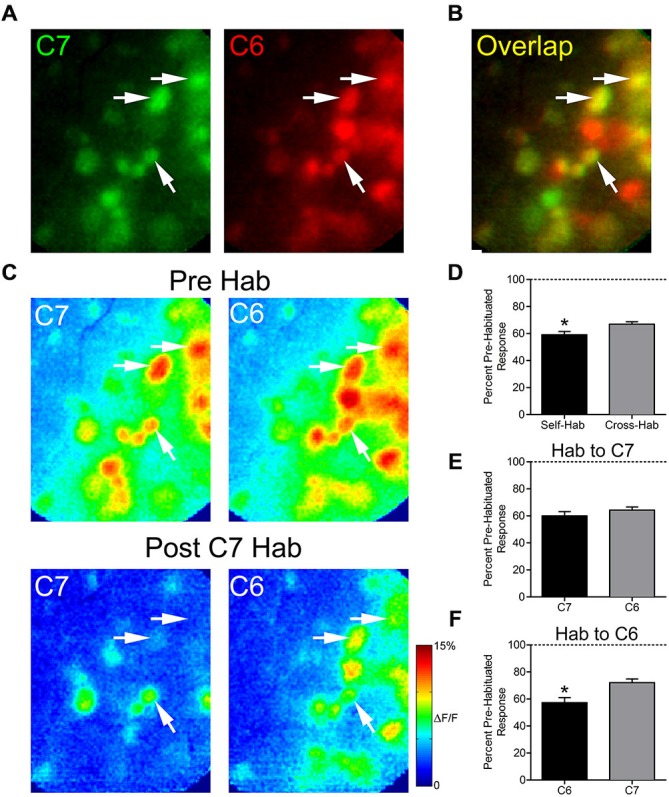
**Thirty seconds odor exposure decreases subsequent glomerular responses to a structurally similar odor. (A)** Baseline glomerular responses to 2-heptanone (C7) and 2-hexanone (C6) in the same animal displayed in different color channels (C7, 0.5% s.v.: green; C6, 0.5% s.v.: red) at 10× magnification. **(B)** Overlay of the baseline glomerular responses to C7 and C6 shown in **(A)**, highlighting glomeruli (yellow) that respond to both odors. White arrows indicate some examples of these shared glomeruli. **(C)** Pseudo-color glomerular responses to C7 and C6 before (Pre Hab) and after (Post C7 Hab) a 30-s exposure to C7. For both odors, glomerular responses are decreased from their baseline following the habituating odor exposure. **(D)** Mean normalized glomerular responses to both the habituated odor and the cross-habituated odor are reduced. Habituated responses are significantly lower than cross-habituated responses. **(E)** When C7 was used as the habituating odor, self- and cross-habituated responses were not significantly different. **(F)** When C6 was used as the habituating odor, self-habituated responses were significantly lower than cross-habituated responses. Error bars indicate SEM. *p < 0.05.

We next evaluated whether prolonged exposure to an odor would affect the subsequent glomerular response to a structurally similar odor, an effect known as cross-habituation (Wilson, 2000). In five animals, pre-habituation baseline responses to 2-hexanone (C6) and 2-heptanone (C7) were established (Figure 3A). These odors differ by only a single carbon and share some activated glomeruli (Figure 3B). We assessed the effects of both self- and cross-habituation in shared glomeruli (Figure 3C). Pooling all habituation trials, regardless of habituating odor, shared glomeruli showed reduced mean normalized responses to both the habituated odor (59.1 ± 2.4% of baseline) and to the cross-habituated odor (66.9 ± 1.8% of baseline) with responses to the habituated odor significantly lower than those to the cross-habituated odor (paired *t*-test: *t*_(61)_ = 2.53, *p* < 0.05, *n* = 62 glomeruli; Figure 3D). When the longer carbon chain odorant, C7, was used as the habituating odor, the cross-habituation (response to C6: 64.3 ± 2.4% of baseline) was not different from self-habituation (response to C7: 60.1 ± 3.1% of baseline; paired *t*-test: *t*_(40)_ = 0.97, *p* = 0.33, *n* = 41 glomeruli; Figure 3E). However, when the shorter carbon chain odorant, C6, was used as the habituating odor, the cross-habituation (response to C7: 72.1 ± 2.7% of baseline) was significantly less than the self-habituation (response to C6: 57.3 ± 3.8% of baseline; paired *t*-test: *t*_(20)_ = 4.92, *p* < 0.0001, *n* = 21 glomeruli; Figure 3F).

**Figure 4:**
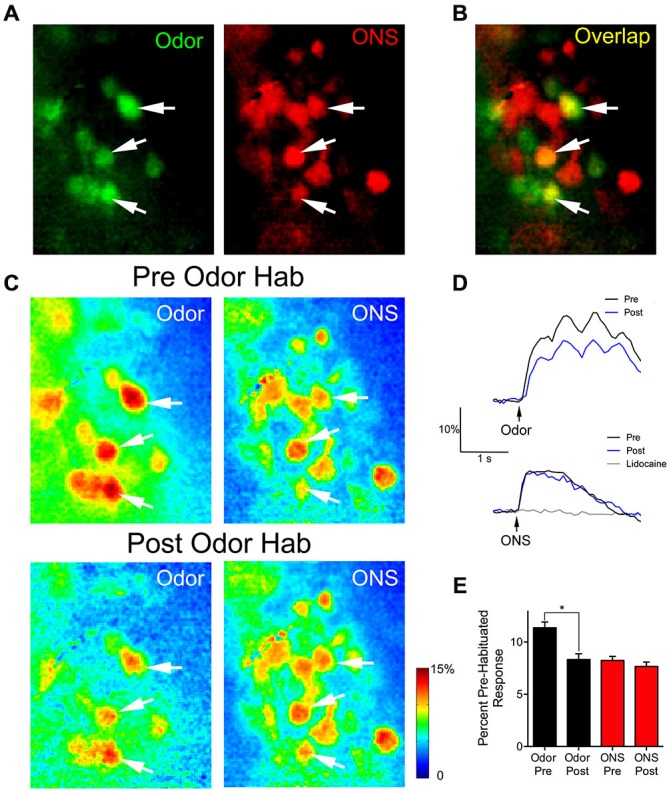
**Thirty seconds odor exposure decreases subsequent glomerular responses to odors, but not to ON electrical stimulation. (A)** Baseline glomerular responses to an odor presentation and ON electrical stimulation (ONS) in the same animal displayed in different color channels (2-heptanone, 0.5% s.v.: green; ONS, 100 μA: red) at 10× magnification. **(B)** Overlay of the baseline glomerular responses to odor and ONS shown in **(A)**, highlighting glomeruli (yellow) that respond to both stimuli. White arrows indicate some examples of these shared glomeruli. **(C)** Pseudo-color glomerular responses to odor and ONS before (Pre Odor Hab) and after (Post Odor Hab) a 30-s exposure to 2-heptanone. Thirty seconds after a habituating odor exposure (bottom panel), glomerular responses to 2-heptanone are significantly decreased compared to control. However, 1 min after the habituating odor exposure, glomerular responses to ON stimulation are unchanged. **(D)** Example fluorescence traces taken from an overlapping glomerulus (**A–C**: middle white arrow) responding to both 2-heptanone (top panel) and ONS (bottom panel) before (black trace) and after (blue trace) odor habituation. The gray trace in the bottom panel shows the response to ONS following bulbar lidocaine application. Black arrows indicate stimulus onset. **(E)** Population data show glomerular responses to the odor were significantly reduced following odor habituation, while pre and post ONS responses in the same glomeruli were unchanged. Error bars indicate SEM. *p < 0.05.

We used olfactory nerve-stimulation (ONS) to assess whether reduced glomerular responses following prolonged odor stimulation reflect synaptic depression of OSN input. To accomplish this, we stimulated the axons of the OSNs within the OB to generate glomerular responses without odorant activation. In two animals, responses to ONS were compared before and after OB lidocaine application to verify that ONS was not directly activating glomeruli (Figure 4D; gray trace). Following bulbar lidocaine application, glomerular responses to ONS were completely blocked (Pre: 7.0 ± 0.3% ΔF/F; Post: 0.3 ± 0.2% ΔF/F; one sample *t*-test: *t*_(21)_ = 1.85, *p* = 0.09, *n* = 22 glomeruli). In four animals, pre-habituation baseline responses to one of the odors and to electrical ONS were established (Figure 4A). Analysis was performed on overlapping glomeruli that were activated by both the odor and the ON stimulation (*n* = 28; Figure 4B). Glomerular responses changed within 1 min following the odor habituation trial (ANOVA: *F*_(3,81)_ = 21.25, *p* < 0.0001). Post hoc tests showed significant reduction of the mean glomerular response to odor (Pre: 11.4 ± 0.6% ΔF/F; Post: 8.3 ± 0.5% ΔF/F; Figures 4C–E). However, in the same glomeruli, the mean glomerular response to ONS was not significantly reduced following odor habituation (Pre: 8.2 ± 0.4% ΔF/F; Post: 7.7 ± 0.4% ΔF/F), demonstrating that postsynaptic responses independent of odor input were not depressed.

## Discussion

We imaged excitatory postsynaptic glomerular odor responses and observed reductions in glomerular responsiveness following prolonged odor stimulation. Glomerular responses to an odor were decreased following exposure to both the same odorant (self-habituation) and a structurally similar odorant (cross-habituation). ONS following odor habituation showed that these decreases were not a result of OSN-M/T cell synaptic adaptation and suggests that reduced glomerular responses reflect processes taking place in the periphery.

Decreased OB activity following prolonged odor stimulation has been observed with multiple recording modalities (Potter and Chorover, 1976; Chaput and Panhuber, 1982; Wilson, 2000; McKeegan and Lippens, 2003; Schafer et al., 2005; Chaudhury et al., 2010). An fMRI study in anesthetized rats found that the glomerular layer showed significantly decreased BOLD signal responses to subsequent odor exposures for up to 5 min following a 32-s odor presentation (Schafer et al., 2005). Electrophysiological recordings of single M/T cell odor responses in anesthetized rats (Wilson, 2000; Fletcher and Wilson, 2003) showed a similar amount of habituation and recovery time of several minutes. Our results fit well with these studies and demonstrate that reduced excitatory odor responses following habituation can be seen at the earliest stages of OB response and are propagated through the OB relatively unchanged.

Our finding of significant cross-habituation at the glomerular population level is similar to previous electrophysiological studies in anesthetized rats that showed single-unit M/T cell responses to other structurally similar odors within their receptive field are also significantly decreased following prolonged exposure to an odor (Wilson, 2000; Fletcher and Wilson, 2003; Chaudhury et al., 2010). Overall, we found that self-habituation results in a larger reduction of the glomerular response than cross-habituation. However, further analysis showed that the effects of cross-habituation are asymmetrical. While the magnitude of self- and cross-habituation are the same after prolonged exposure to the longer carbon chain odorant, after exposure to the shorter chain odorant the magnitude of cross-habituation is significantly less than that of self-habituation. Asymmetrical effects have been observed in the OB (Wilson, 2000), and even perceptually in humans (Cain, 1970). While still unexplained, the asymmetry could reflect the fact that odorants of increasing carbon chain length activate increasing percentages of OSNs (Malnic et al., 1999). In our case, if C6 is unable to activate as many OSNs as C7, then there is a higher likelihood that there will be un-habituated C7 neurons after an exposure to C6, resulting in less cross-habituation magnitude at the glomerular layer.

ON-stimulation allowed us to test whether glomerular habituation still occurs in the absence of epithelial OSN activation. A similar method was used to explore the effects of odor habituation on synaptic efficiency at the M/T cell-piriform cortex pyramidal neuron synapse (Wilson, 1998). Interestingly, we found that, after prolonged odor exposure, glomeruli had decreased responses to odor, but showed no significant decreases in their response to ON-stimulation. These results indicate that even though their response to odor is decreased following prolonged odor exposure, postsynaptically, the M/T cell dendrites can still be activated and presynaptically, glutamate is available and able to be released effectively from the OSN terminals (i.e., adaptation is likely occurring distal to the ON layer). Lecoq et al. (2009) found evidence that fast adaptation of the glomerular odor response during high-concentration odor stimulation in anesthetized rats is also peripherally mediated. Together, these results suggest that OB glomerular habituation at the timescale of our experiments is mediated by peripheral OSN adaptation and does not heavily rely on synaptic depression of OSN input or further processing via bulbar circuits.

In contrast to our findings, some studies have demonstrated that recovery from adaptation takes place faster in the periphery than in the OB (Potter and Chorover, 1976; Schafer et al., 2005), indicating that additional bulb circuitry was involved. However, in our study M/T glomerular response seems relatively unaffected by bulb processes. This could be due to differences in methodology, since the prior studies used either longer (e.g., 10 min) or repeated (e.g., 10 × 30 s) odor presentations. Because they utilized more intensive odor stimulation, these studies might reflect bulbar depression mechanisms uncovered by studies which used protocols involving repeated (Chaudhury et al., 2010) or much longer (Larkin et al., 2010; Das et al., 2011; Ramaswami, 2014) odor presentations.

Peripheral olfactory adaptation is complex and still not well understood, however, several possible mechanisms have been outlined (Zufall and Leinders-Zufall, 2000; Reisert and Zhao, 2011). Studies have indicated that the gaseous signaling molecules, carbon monoxide and nitric oxide, play a role in OSN adaptation that has been shown to last for several minutes (Zufall and Leinders-Zufall, 1997, 1998; Brunert et al., 2009). It has been postulated that these messengers could be important not only for habituation, but for cross-habituation as well, since they are able to diffuse across the nasal epithelium and potentially affect others OSNs (Brunert et al., 2009). While our experiments did not allow us to probe the specific peripheral adaptation processes underlying the decreased glomerular responses, if the OSNs synapsing onto the glomeruli we observed were adapted in this manner, it could explain the relatively subtle, but longer-lasting decrements we recorded.

In conclusion, the present study found that glomerular responses to odors are decreased following a habituation trial, however, our ON-stimulation experiment showed that this reduction seems to reflect uniform distal adaptation of OSNs, rather than transmitter rundown at the glomerular synapse or depression of bulb circuits. Intriguingly, this indicates that though input to the glomerular layer has been reduced, it can still be activated, should contingencies change. The OB, including the glomerular layer, receives cortical feedback (Brunjes et al., 2005; Boyd et al., 2012; Markopoulos et al., 2012) as well as cholinergic, noradrenergic, and serotonergic input (Fletcher and Chen, 2010), all of which have been shown to modulate OB responsivity (Petzold et al., 2009; Ma and Luo, 2012; Eckmeier and Shea, 2014; Rothermel et al., 2014). Future experiments should probe the potential of these centrifugal inputs to affect OB habituation.

## Funding

This research was supported by the Pew Biomedical Science Scholars Program and by the National Institutes of Health Grant DC013779 to MLF.

## Conflict of Interest Statement

The authors declare that the research was conducted in the absence of any commercial or financial relationships that could be construed as a potential conflict of interest.
